# Family aggregation of the Intelligence Quotient: understanding its role in first episode of psychosis

**DOI:** 10.1192/j.eurpsy.2022.320

**Published:** 2022-09-01

**Authors:** N. Murillo-García, J. Soler, M. Fatjó-Vilas, R. Ayesa-Arriola

**Affiliations:** 1 IDIVAL, Valdecilla Biomedical Research, Research Group On Mental Illnesses, Santander, Spain; 2 University of Barcelona, Department Of Evolutionary Biology, Ecology And Environmental Sciences, Barcelona, Spain; 3 CIBERSAM, Center for Biomedical Research in Mental Health Network, Mental Health, Madrid, Spain; 4 Valdecilla Biomedical Research Institute, Psychiatry, Santander, Spain

**Keywords:** Familial aggregation, Intelligence Quotient, Neurocognition, First episode of psychosis

## Abstract

**Introduction:**

The familiality of intelligence quotient (IQ), understood as its similarity among family members, might be related to different manifestations in first episode of psychosis (FEP) patients.

**Objectives:**

To estimate the IQ familiality through the intra-family resemblance score (IRS) in FEP patients and their unaffected first-degree relatives; and to analyze if the deviation from the family-IQ is related to the patients’ premorbid, clinical and cognitive characteristics.

**Methods:**

Individuals from 129 families participated in this study (129 patients, 143 parents, 97 siblings). For each family, two values were estimated: the family-IQ, obtained by the mean IQ of the patient and his/her relatives (using the WAIS vocabulary subtest); and the IRS, an index previously reported that indicate intra-family heterogeneity (IRS<0) or homogeneity (IRS>0) for a given trait. According to the IRS and the family-IQ, patients were assigned to 6 groups (Figure 1).

**Results:**

FEP patients in families with heterogeneous IQ (IRS<0) had a significantly lower IQ than their relatives (p<0.001). Also, those with low IQ and from heterogeneous families had poorer childhood adjustment (p=0.001). The patients with high IQ belonging to homogenous families showed less positive symptoms at baseline (p=0.009). FEP patients in homogenous families due to low IQ evidenced the lowest neuropsychological performance (Figure 2).

**Figure:**
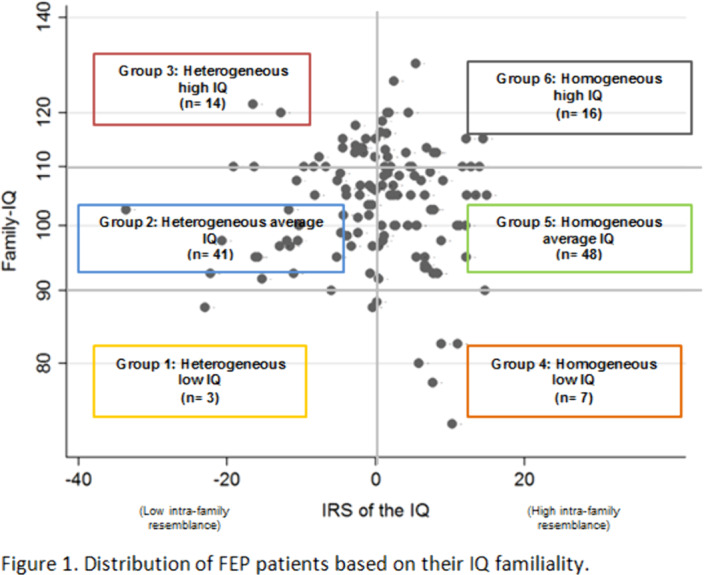

**Figure:**
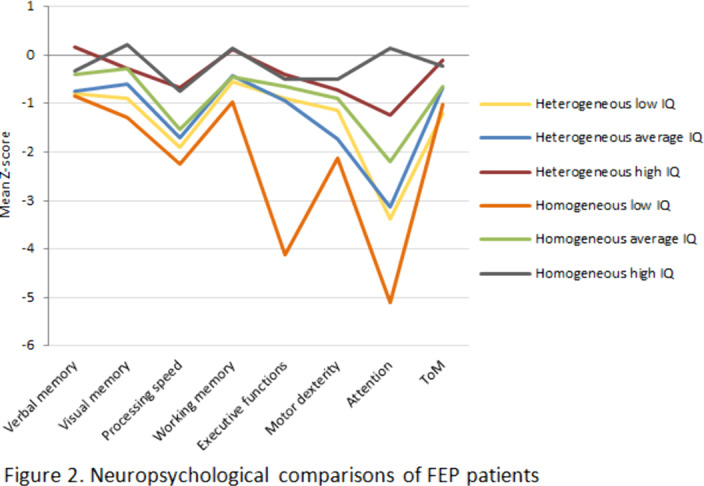

**Conclusions:**

The analysis of the IQ familiality and the concordance/discordance of the patients’ and relatives’ IQ, offers a new approach for the characterization of different premorbid, clinical and cognitive profiles in FEP patients. The relationship between deviation from the family-IQ and poor premorbid childhood adjustment supports the neurodevelopmental hypothesis of schizophrenia.

**Disclosure:**

No significant relationships.

